# Supplementary figures and images for: Sleep drive reconfigures wake-promoting clock circuitry to regulate adaptive behavior
Source: PLoS Biol. 2021 Jun 30;19(6):e3001324. doi: 10.1371/journal.pbio.3001324 (PMC8277072; doi:10.1371/journal.pbio.3001324)

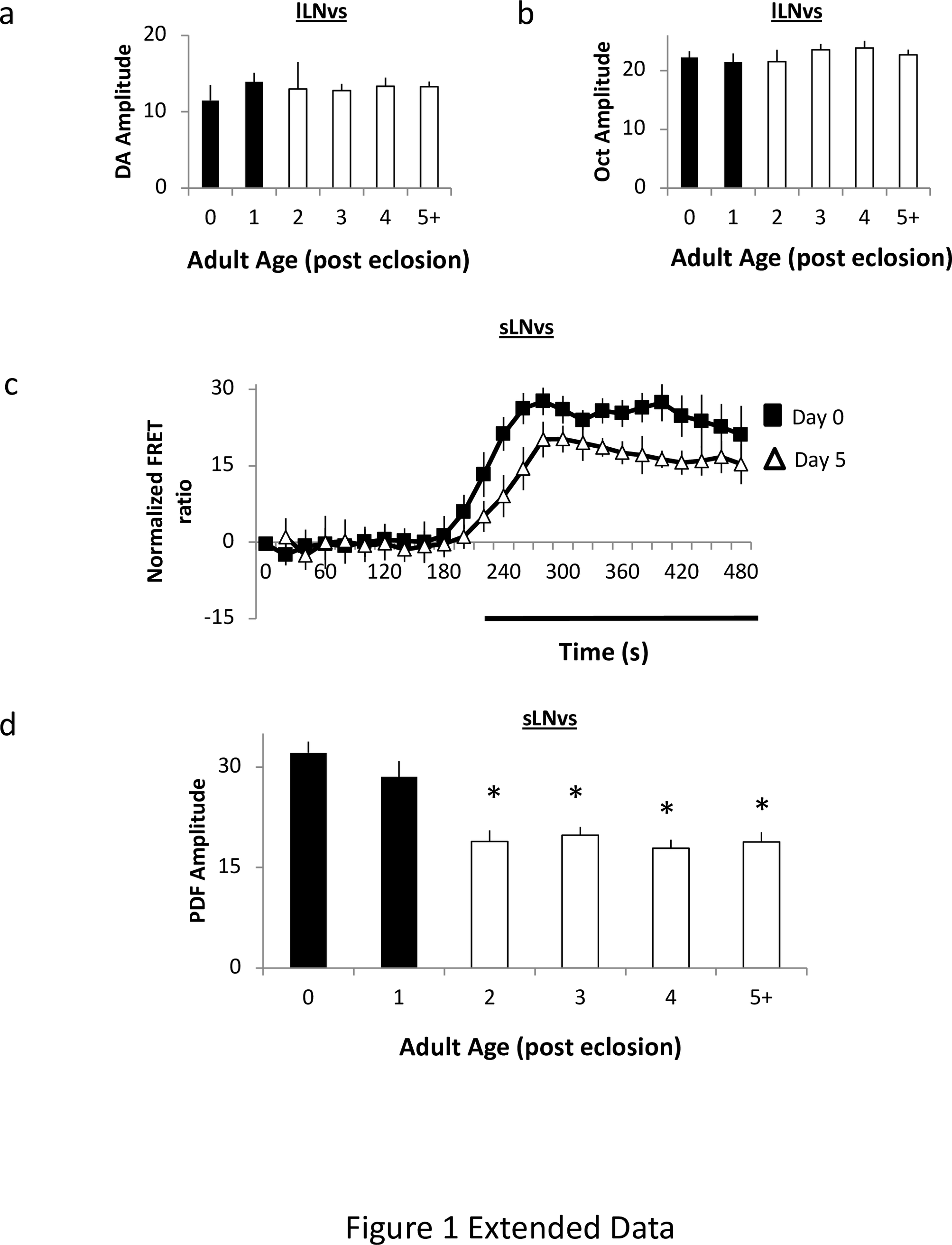

Supplement: S1 Fig — (A, B) Response of l-LNvs to DA and Oa in Pdf-GAL4>UAS-Epac1 flies from day 0 to day 5+ (n = 4–15 hemi-segments per age, ANOVA F[5,36] = 6.08, p = 0.96 and ANOVA F[5,54] = 8.93, p = 0.90, respectively). (C) Normalized FRET ratio in s-LNvs before and during PDF exposure on day 0 (n = 6) and day 5 (n = 7). (D) PDF response amplitude in s-LNvs on day 0 to day 5+ (ANOVA F[5,76] = 13.31, p = 3.75E-9 n = 8–20 hemi-segments per age). *p < 0.05, modified Bonferroni test. Data underlying this figure can be found in S7 Data. DA, dopamine; FRET, Förster Resonance Energy Transfer; l-LNv, large ventral lateral neuron; Oa, octopamine; PDF, pigment dispersing factor; s-LNv, small ventral lateral neuron. (TIF) [file pbio.3001324.s001.tif]

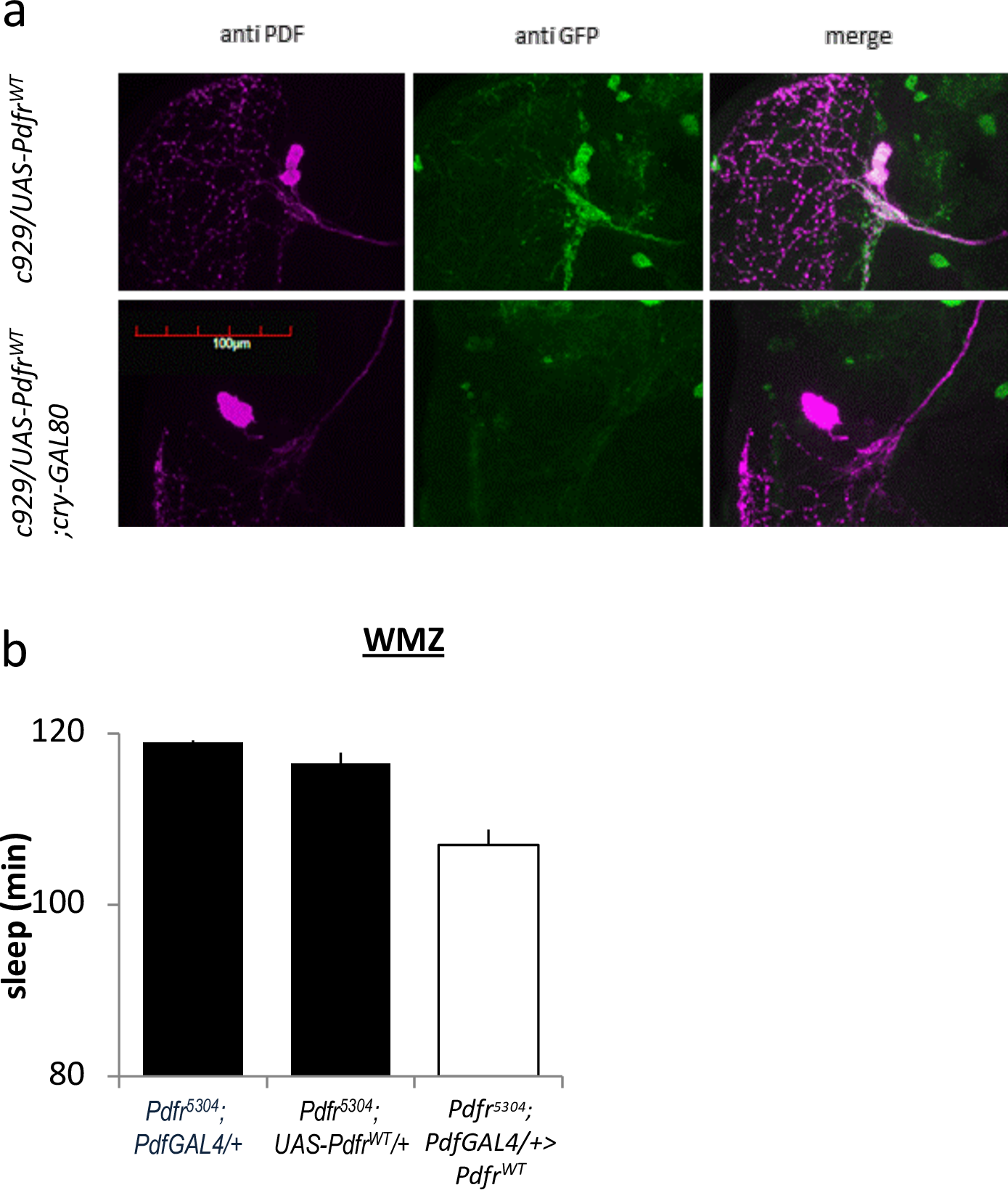

Supplement: S2 Fig — (A) Immunohistochemistry for PDF and GFP reveals the expression of GFP in the l-LNvs of c929-GAL4/UAS-gfp flies but not in the brains of c929-GAL4/UAS-gfp; Cry-Gal80 flies. (B) Pdfr5504; PDF>/UAS-PdfrWT flies exhibit more waking during the WMZ than Pdfr5304; Pdf-GAL4/+ and Pdfr5304;UAS-PdfrWT/+ parental controls (ANOVA F[2,91] = 4.63, p = 0.01 n = 41–64 flies/genotype) flies. Data underlying this figure can be found in S8 Data. GFP, green fluorescent protein; l-LNv, large ventral lateral neuron; PDF, pigment dispersing factor; WMZ, wake maintenance zone. (TIF) [file pbio.3001324.s002.tif]

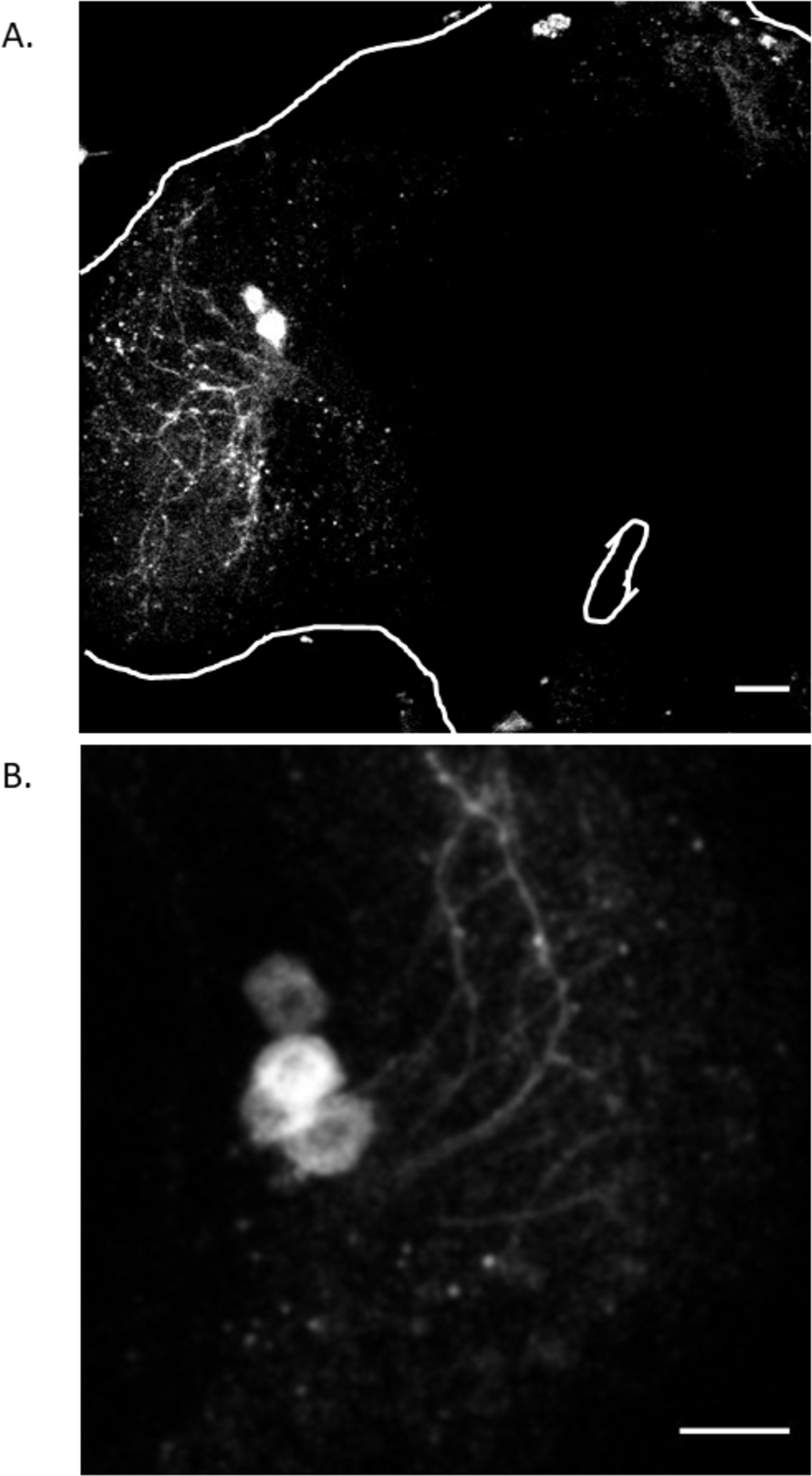

Supplement: S3 Fig — (A) CNS with overlay traced reveals cell bodies and optic lobe projections of l-LNvs in the left hemi-segment of a brain. (B) Four cell bodies and projections of l-LNvs of right hemi-segment. Z-stack projections with 2 μm steps. Scale bar: 15 μm. GFP, green fluorescent protein; l-LNv, large ventral lateral neuron. (TIF) [file pbio.3001324.s003.tif]

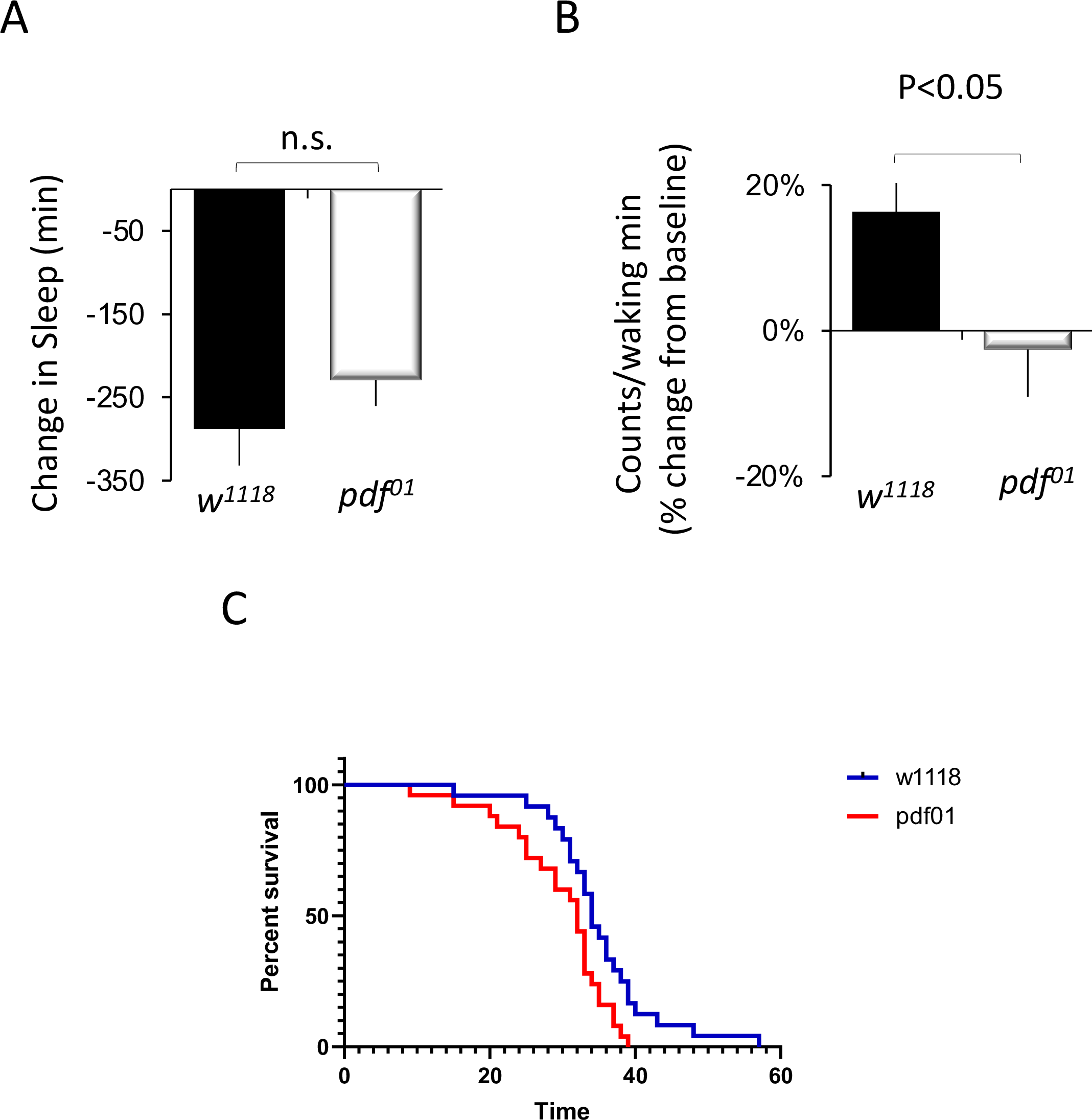

Supplement: S4 Fig — (A) Sleep was reduced in both pdf01 mutants and w1118 genetic controls during the first 18 h of starvation (data presented as change from baseline; (n = 20–22 flies/genotype, p > 0.05). (B) During the first 18 h of starvation, waking activity was significantly lower in pdf01 mutants compared to w1118 controls (p < 0.05). (C) Kaplan–Meier analysis reveals % survival during starvation in pdf01 (n = 25) flies and w1118 (n = 24) controls (χ2 = 6.20, df = 1, p = 0.01). Data underlying this figure can be found in S9 Data. n.s., not significant. (TIF) [file pbio.3001324.s004.tif]

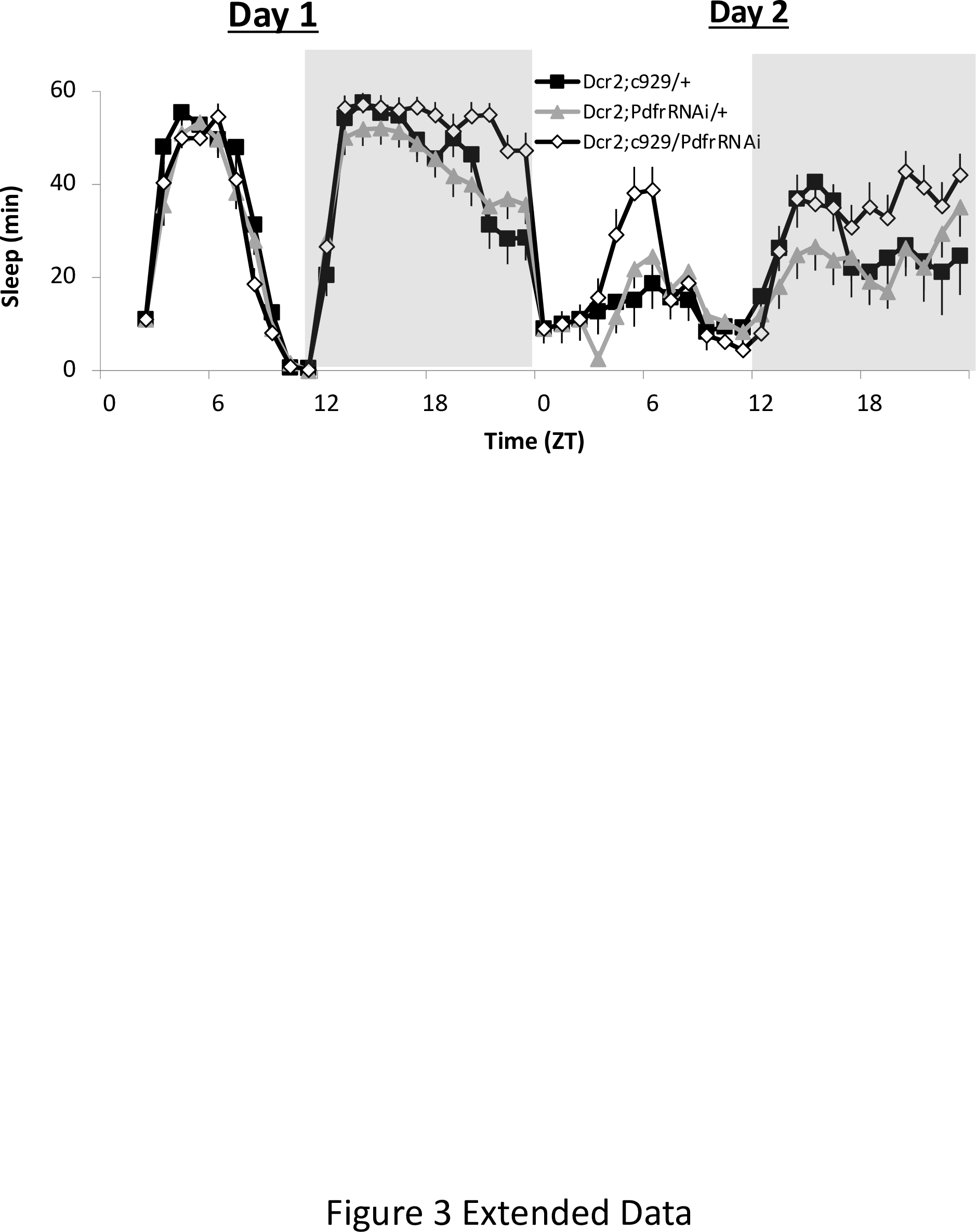

Supplement: S5 Fig — (A) Sleep (minutes) during 48 h of starvation in Dcr2; c929-GAL4/UAS-pdfrRNAi flies (n = 24), Dcr2; c929-GAL4/+ (n = 18), and +/UAS-PdfrRNAi (n = 32) control flies. ZT, Zeitgeber time. (TIF) [file pbio.3001324.s005.tif]

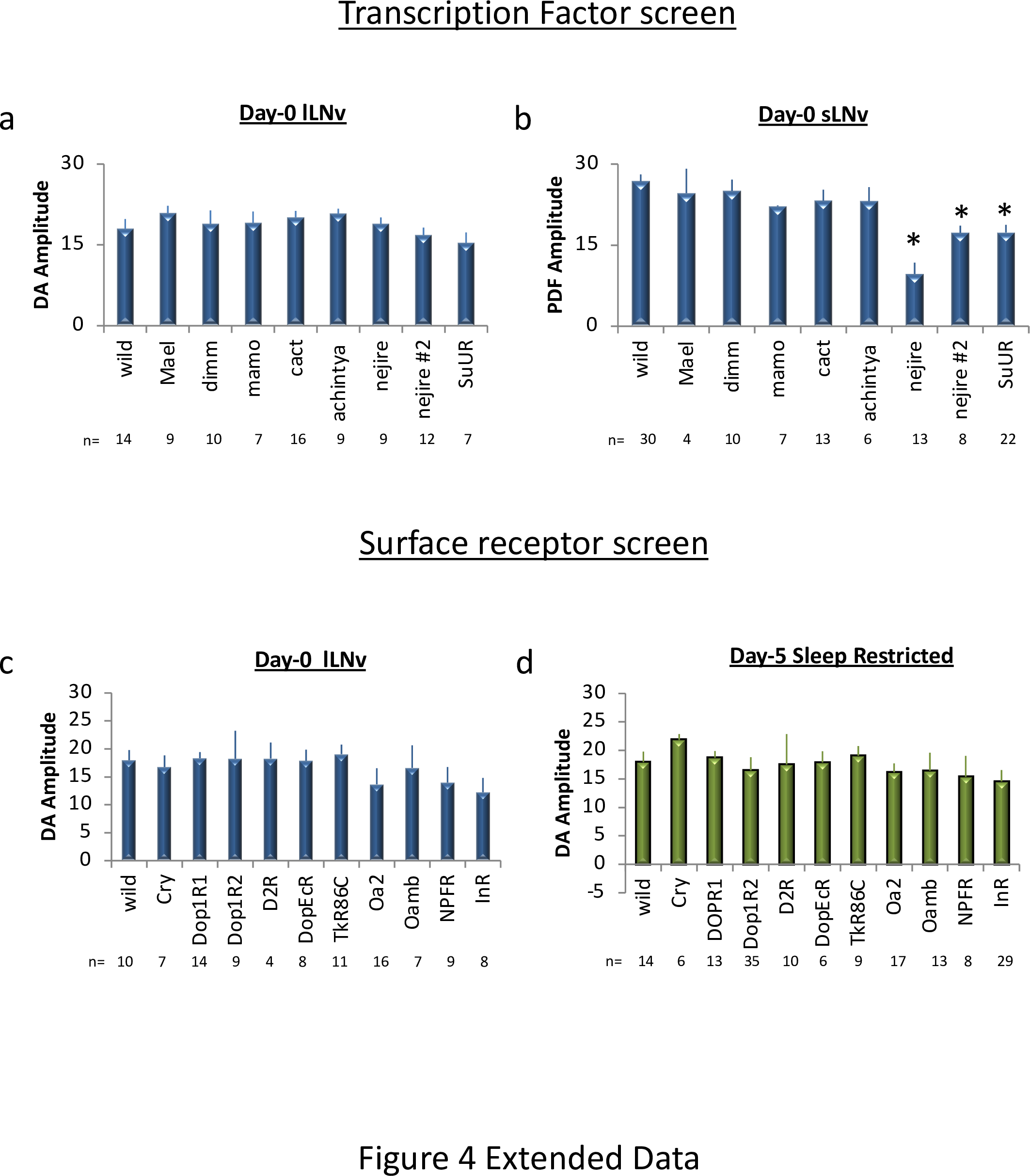

Supplement: S6 Fig — (A) The amplitude of l-LNv responses to DA on day 0 in Pdf-GAL4>UAS-Epac1 flies coexpressing RNAi lines for the depicted transcription factors (ANOVA F[8,92] = 1.04, p = 0.42; n is as indicated beneath each bin). (B) The amplitude of s-LNvs responses in Pdf-GAL4>UAS-Epac1 flies coexpressing RNAi lines for the depicted transcription factors neurons on day 0 (ANOVA F[8,112] = 9.36, p = 1.19E-9). (C) The amplitude of s-LNvs responses to DA on day 0 in Pdf-GAL4>UAS-Epac1 flies coexpressing RNAi lines for the depicted cell surface receptors (ANOVA F[10,108] = 0.79, p = 0.63; n is as indicated beneath each bin). (D) The amplitude of l-LNvs responses to DA following sleep restriction in 5-day-old Pdf-GAL4>UAS-Epac1 flies coexpressing RNAi lines for the depicted cell surface receptors (ANOVA F[10,159] = 0.42, p = 0.94; n is as indicated beneath each bin). Data underlying this figure can be found in S9 Data. DA, dopamine; l-LNv, large ventral lateral neuron; PDF, pigment dispersing factor; RNAi, RNA interference; s-LNv, small ventral lateral neuron. (TIF) [file pbio.3001324.s006.tif]
