# Supplementary material for: Sleep drive reconfigures wake-promoting clock circuitry to regulate adaptive behavior
Source: PLoS Biol. 2021 Jun 30;19(6):e3001324. doi: 10.1371/journal.pbio.3001324 (PMC8277072; doi:10.1371/journal.pbio.3001324)
Supplement: Attachment — Submitted filename: Klose and Shaw Response to Reviewers feb 24.docx [file pbio.3001324.s017.docx]

Reviewer #1: In this study, Klose and Shaw describe a striking phenomenon in which an important GPCR for circadian timekeeping (PdfR) is expressed transiently within wake promoting clock neurons (the l-LNvs) during early adulthood to promote late day wakefulness during a developmental window characterized by high levels of sleep.  Remarkably, they show that the expression of PdfR is reinstated during times of extended sleep disruption.  The authors also examine the fitness implications for this phenomenon and address the relationships between sleep disruption and l-LNv PdfR expression in detail.  They also go on to implicate genes mediating the context dependency of PdfR expression in the l-LNvs.  These results are highly significant and of great interest.  The following points, all modest or minor, might be considered by the authors to improve this very interesting contribution to the field:

1.)     What about the PDF neuropeptide?  PdfR is a type II GPCR, a class of receptors known for their promiscuity.  Are the effects of PdfR signaling in the l-LNvs during times of high juvenile sleep drive and during prolonged sleep disruption mediating PDF signals or signals from some other peptide?  It's surprising not to see the Han5304 experiments repeated for Pdf01 mutants.  The results of this analysis would be interesting either way and the central conclusion would not depend on the outcome, but the absence of Pdf01 data here produces a bit of an itch that could be easily scratched.

We appreciate how using the Pdf01 mutant might be helpful. However, in our experience, scratching the Pdf01 mutant itch to assess/confirm/explore Han5304 phenotypes has proven to be frustrating. We would like to note that we evaluated the Han5304 mutant in isolation only as proof of principle so that we could then evaluate phenotypes upon rescue. In any event, the literature indicates that the PDF01 and Han5304 mutant data don’t always line up well.

2.)     The authors should provide a more detailed rationale for focusing on the 2-h time-window just before dusk as a time corresponding "high sleep-drive."  This might confuse some readers, as this is the window in which flies show anticipatory evening activity.  Readers might therefore be predisposed to expect low sleep drive here.  The idea that this is specific to sleepy young flies could be clarified.

We apologize for the confusion. We agree that it can be difficult to disassociate high-wake drive from low-sleep drive. However, it is possible to increase sleep drive prior to lights-off by keeping flies awake for 22 hours from 8pm till 6pm the next day. It was this type of experiment that was used to identify the wake-maintenance zone in mammals and in flies. Our previous studies have shown that 12 h of nighttime sleep deprivation ending at lights-on results in a large sleep rebound in adult flies. However, when adult flies are deprived of sleep for an additional 10 h and released into recovery at 6pm, rebound is not typically observed until the next morning. We have modified the text to clarify this relationship.

During the day, sleep is highest during the mid-day siesta and is reduced in the hours preceding lights out, when anticipation of the impending shift from light to dark is demarcated by increased waking activity (Fig. 1a) (11, 17, 18). We have operationally defined the 2 h period before lights out as the wake-maintenance zone based upon the observation that sleep rebound is absent or dramatically reduced when  flies are released into recovery during this time window (17, 27). The ability to maintain waking during this special time window in the face of increased sleep-drive resulting from sleep disruption suggests that this window of time is protected for important waking behaviors (28).

3.)     The description of the methods for gauging mating success should be more detailed. Without more detail is it difficult to gauge the quality of the experimental design and the appropriateness of the statistical analysis.

We apologize for the lack of detail. We have added the following description to the Method section:

Mating success assay consisted of putting one virgin female in a vial with a single male of the genotype to be evaluated. Each pair of flies was placed into a vial at ZT4 on the day of eclosion and then the male was removed at ZT24. Mating success was determined days later through visual inspection of viable offspring (pupae, larvae, etc.). Females from vials that produced no offspring were subsequently provided several males to test for her reproduction viability.

4.)     There are no error bars shown on figure depicting mating success data (3A-C), where these experiments replicated?

These experiments were replicated. The replicates were combined and the graphs depict the % of flies that produced offspring. We now include the chi-square results in the figure legend which now reads

(A) Cs flies produce more offspring than Pdfr5504 mutants (n=30/genotype); χ2= 12.13, p=.0004. (B) Dcr2; c929-GAL4/UAS-PdfrRNAi flies produce fewer vials with offspring compared to Dcr2; c929-GAL4/+ and +/UAS-PdfrRNAi parental controls; χ2= 21.3, p=.00002 (C) Pdfr5504; c92-GAL4; UAS-PdfrWT (rescue, green) flies produce more offspring than Pdfr5504; UAS-PdfrWT/+ (purple), Pdfr5504; c929-GAL4/+; CryGal80/+ (red), and Pdfr5504; c929-GAL4/UAS-Pdfr; Cry-Gal80 (blue) parental controls (n=30/genotype) χ2= 8.95, p=.029.

5.)     Although the survival results depicted in Figs. 4C and D are quite striking and clear, a Kaplan-Meier survival analysis should be done for the statistical analysis on these data.

We thank the Reviewer for this suggestion. We have analyzed the data using the Kaplan-Meier survival analysis and have included created new figures 4C and 4D with the corresponding statistic in the figure legend.

6.)     In Fig. 5D, the baseline sleep has clear MYC signal, which is not is what is reported in the results.  Is this a labeling error (i.e., are PDF and and MYC labels switched)?

We thank reviewer #1 for noticing this labelling mistake; it has been fixed.

7.)     The data shown in supplemental figure S3 seems critical and should be placed in the main figures (Fig. 5).

We thank the reviewer for this suggestion. We now include the data from supplemental S3 in Figure 5.

8.)     On Page 2, the four citations (10-13) provided for the notion that circadian timekeeping is a distributed network property neglect two important studies at the expense of a review and a study of questionable relevance (Yao et al. 2014 Science Vol. 343, p. 1516 and Yao et al. 2016 Cell Reports Vol. 13, p.2873).

We thank Reviewer #1 for their assistance and agree. We now substitute both references in the text.

9.)     The use of the split GAL4 specific for the l-LNvs is fantastic, but it's used only in a subset of the experiments.  The authors should consider using it alongside all c929-GAL4 experiments, as c929 is widely expressed throughout the brain.

We agree that c929 is expressed throughout the brain. However, the c929 driver has been a work horse of sleep and circadian biology for well over a decade now. When used properly, the c929 driver has proven itself to be accurate and effective. The consistency across all of our experiments, including the rescue experiments, reinforces the notion that the c929 driver is a proven tool to evaluate the role of the lLNvs as they relate to waking. It should be noted that the split-GAL4 line only became available to us after the majority of experiments were complete. Nonetheless, the Split-GAL4 line fully supports the conclusions made with c929-GAL4.

10.)    Page 8, line 11, "factor" should probably be "factors."

We have changed factor to factors

11.)    Page 9, line 1: I don't think it's accurate to call PDF "the main output of the clock" at this point (it also brings the itch of the absence of PDF peptide data back).

We agree with Reviewer #1 and have modified the sentence. It now reads: “In *Drosophila*, PDF differentially coordinates the activity of diverse neuronal groups to optimize behavioral output with the prevailing environmental conditions [14, 48].”

12.)    Page 19, line 2: "~20" should be "~20h."

We now include the time designation.

13.)    I'm not entirely sure about the term "respecification" here.  Might there be a better term or phrase?

We have struggled to find a different term but at this point “respecification” seems to describe the most salient features of the phenotypes we observe. We believe that our description in the Discussion on how this term is similar and different to the way the term is used in the literature will allow the readers to understand why we chose the term. We are also open to using a different term if suggested.

14.)    "Data Table S1 does not match the figures presented in the manuscript.

We have removed Table S1.

15.) The text on the histogram in Fig. 2D is difficult to read.

We have increased the font size and readability of the text in Figure 2D.

Reviewer #2: This is an interesting manuscript that presents evidence for a dynamic expression of Drosophila PDFR in a specific group of circadian neurons - the lLNvs - known to regulate arousal. PDFR expression in adults is limited to the first couple of days following eclosion, when sleep is high.  Also, PDFR expression can be reactivated in case of sleep restriction.  PDFR appears to ensure wakefulness in the evening, and thus improves mating in young adult and survival from starvation.  The last figure of the paper presents a mini-screen for genes that might contribute to PDFR signaling. This part of the manuscript is quite preliminary, but otherwise, overall, this appears to be an important and for the most part solid study.

My main suggestion to improve this manuscript would be to more clearly establish that the genes identified in figure 6 participate in PDFR signaling and sleep regulation.  Do these genes indeed affect sleep similarly to PDFR, do they genetically interact with PDFR?.  Also, I do not understand why downregulation of dopamine receptors decrease PDFR responses, but oddly do not reduce sensitivity of the lLNvs to dopamine.  This needs to be explained.

We show a fair amount of data in Figure 6 that nejire and SuUR participate in PDFR signaling. However, as mentioned by Reviewer #2 we did not link any of the genes evaluated in Figure 6 to sleep. We have now evaluated the impact of expressing *UAS-nej-^RNAi^* and *UAS-nej^WT^* in the l-LNvs using the split-GAL4 driver (SS00645). Unfortunately, the data is not providing a coherent picture. The first issue is that the *l-LNv>UAS-nej^WT^* flies are not healthy and we struggle to get adult flies to test for behavior. Secondly, while both *l-LNv>UAS-nej^WT^* flies and *l-LNv>UAS-Pdfr^WT^* flies increase waking during the day (Revision Figure 1), the increase in waking occur at different times. Specifically, the *l-LNv>UAS-nej^WT^* flies was occurred in the morning and was not confined to the wake-maintenance zone indicating that the impact of nejire extends beyond regulating the expression of PDFR. Thirdly, neither *l-LNv>UAS-nej^WT^ nor l-LNv>UAS-nej^RNAi^* alter survival during starvation unlike Pdfr-^WT^ and Pdfr-^RNAi^. nejire is a large protein that contains several functional domains that can interact with a diverse set of molecular pathways including those that impact metabolism. Thus, it is perhaps not surprising that nejire does not precisely phenocopy the impacts seen for PDFR. Future studies that allow for the expression of the Full Length protein as well as proteins missing various functional domains will help clarify how nejire is regulating PDFR to alter sleep.

While it may seem surprising that the lLNvs continue to respond to DA following the knockdown of individual DA receptors, it should be noted that the l-LNvs express multiple DA receptors and we probed response properties with a high concentration of DA. Importantly our data are consistent with a report that was published while this manuscript was in review (DOI: https://doi.org/10.1523/JNEURO). We now cite this paper in the manuscript. The aforementioned manuscript highlights the complexity and heterogeneity of clock neuron responses to DA. Indeed, while the response to dopamine produces an increase in cAMP levels, the electrical responses of the clock neurons to dopamine appear to differ. These data suggest that the effect of dopamine on the l-LNvs may be context dependent and are influenced by the arousal state of the flies. Teasing apart this complex relationship will be the subject of future investigations.

I also have a couple of concerns about specific experiments:

1)      Is rescue of fig 2b statistically significant? Why is it much weaker than with c929-GAL4? PDF-GAL4 is a strong driver

We apologize for the confusion. The quantification of 2A can be seen in 2C and the quantification of 2B can be found in 2D; they are significantly different during the wake-maintenance zone. We have made this clearer in the figure legend.

To avoid confusion, we do not show any PDF-GAL4 data in Figure 2. In any event, we speculate UAS-pdf-RNAi is not as effective as the global pdfr^5304^ mutant in removing pdfr.

2)      I would suggest that the authors overexpress PDFR in lLNvs of wild-type flies with c929-GAL4 and PDF-GAL4 to ensure that this does not result in gain of functions that could confound the rescue experiments.

Our model predicts that the presence of PDFR in the lLNvs would have a phenotype. Indeed, we show in Figure 4A, that expressing UAS-Pdfr-wt in the lLNvs (using the SS000645 split-GAL4 driver) increases waking during the wake-maintenance zone and reduces survival during starvation as predicted. We have replicated the effect of UAS-Pdfr-wt using pdf^5304^;PDF-GAL4 (Figure S2B) and wild-type Pdf-GAL4 line (data not shown). These results match up with the data presented in Figure 5 which shows that sleep deprivation, sleep restriction and starvation all result in the expression of Pdfr in the lLNvs of adult flies.

3)      Why do control flies sleep so little in the RNAi experiments on fig. 2E?  Are these flies defective for juvenile sleep regulation, or is there considerable variations between genetic backgrounds?  This brings me to wonder how carefully genetic backgrounds are controlled.  Are all flies backcrossed to CS? This was not explained in the material and methods or in the main text.

We apologize that the labels are confusing. We are only looking at sleep during the last two hours of the day during the Wake Maintenance Zone. We had hoped that the title above the graph would make that clear but the Y-axis is simply labeled sleep (min). We have now changed the y-axis labels to emphasize that the amount of sleep reported is only for a portion of the day.

Minor comments:

1)      Is "receptor respecification" a commonly used term?  It would be helpful to define this as it is used throughout the manuscript

The term receptor respecification is commonly used. Even colloquially, the term is accurate with respect to our observations: “To specify again; to alter an earlier specification”

The original mention of the term respecification is found in the abstract. We have now modified the sentence to define respecification as the “activity-dependent addition of cell-surface receptors”. In addition, we reduced our use of the word throughout the manuscript.

2)      The first sentence of the last paragraph of p.2 should be rephrased, as it sounds as if sleep is an environmental cues.  It is also not clear to me how sleep synchronizes central and peripheral clocks

We thank Reviewer #2 for catching this awkward phrasing. We have modified the sentence, it reads: Central and peripheral clocks can be synchronized by sleep and environmental cues such as light, temperature, food, social and interactions.

4)      The first sentence of p.3 needs references

We thank Reviewer 2 for catching this. We now include the following references PMID: 28552314 and PMID: 26917772

5)      My understanding is that only about half of circadian neurons express PDFR, based on work by the Shafer and Taghert labs (Im et al., 2010, 2011 and Yao and Shafer 2014).  The relevant statement at the end of the introduction should be corrected.

We agree. The sentence now reads: PDF is expressed in both the large and small ventral lateral neurons (l-LNvs and s-LNvs). In contrast to other clock neurons, the l-LNvs are not believed to express PDFR [13, 27-29].

6)      The H on figure 1 is missing.

We thank Reviewer #2 for catching this; we have inserted the letter H in to Figure 1.

7)      I would indicate on page 4 that there is a noticeable reduction in PDFR sensitivity in sLNv

We state that: Finally, we examined an adjacent group of clock neurons, the small ventrolateral neurons (sLNv) [34]. Responses to PDF in sLNvs are present at the beginning of adulthood, and then decrease in amplitude over the first ~48 hrs of adulthood.

8)      Cry-gal80 is only expressed in CRY+ neurons.  The relevant statement at the bottom of p.4 needs to be corrected.

We thank reviewer #2 for the suggestion. The new sentence reads: “Since c929-GAL4 is expressed in other peptidergic neurons [37] we combined c929-GAL4 with cry-Gal80, which targets the GAL4 inhibitor GAL80 to all CRY+ clock neurons including the lLNv neurons to suppress the PDFR rescue”

9)      On p.4, the authors say that they hypothesized PDFR would be there to promote wakefulness in the evening.  While indeed this was the case, the authors could explain better why they thought this would be the case.  It seems to me PDFR could have been there to promote sleep.

The hypothesis was based, in large part, on the fact that the lLNvs promote wakefulness. Since we initiated these experiments it has become increasingly clear that specific ligands have multiple and divergent roles in different circuits. After reviewing the sentence again, we feel that the focus is more on the wake-promoting impact of the l-LNvs rather than that of PDFR.

10)     Panels shown on figure 3 are also present on figure S2

We have removed the duplicate figure

11)     Second paragraph, p.5, correct "PDF" to "PDFR"

We thank Reviewer 2 for noticing this; it has been fixed.

12)     Figure 5E. PDf amplitude is not a clear label for the y-axis.  Also, it would be clearer to spell out day 1 and day 2 rather then just having 1 or 2 on the x-axis

We agree with these suggestions. We have changed the y-axis label to normalized FRET ratio, to be consistent with the other figures. In addition we have spelled out day-1 and Day-2 on the x-axis.

Reviewer #3: The authors present convincing data that Pdfr expression and receptivity to PDF changes with age in the large LNv, and that perturbations in adult sleep or feeding can induce Pdfr expression in the lLNv. The authors also find that the behavioral effects of loss of Pdfr appear different in young flies than phenotypes previously reported in older adults.

Major Comments:

1.      It's not clear whether the physiologically relevant changes in PDFR expression/ PDFR signaling are mainly limited to the large LNv or if other cell groups may contribute. The authors rely primarily on C929-GAL4 to argue for function in the lLNv group, but C929 is expressed outside of the lLNv. The use of cryGAL80 with C929 is helpful but may not limit function to the lLNv given that it blocks expression in other clock neuron groups. Instead, pdfGAL80 would be a much cleaner reagent.

As mentioned above, the lLNv split-GAL4 driver produced results consistent with that observed for c929-GAL4 driver in that they both increase waking during the wake-maintenance zone. As mentioned in response to Reviewer #2, we have also found that expressing UAS-Pdfr-wt using Pdf-GAL4 and *pdf4r^5304^;Pdf-GAL4* results in increased waking during the wake-maintenance zone similar to both c929-GAL4 and SS000645-splitGAL4. Together with the observation that coupling cryGAL80 with C929 is a common and historically successful tool to evaluate waking, our results implicate the lLNvs. Identifying other neurons that add the PDFR during high sleep drive is of interest and will be addressed in future studies.

2.      In addition, the authors describe a split driver that is specific to the lLNv—the expression pattern and specificity of this line should be documented and functional analysis performed to confirm pdfr function in the lLNv.

We obtained the split-GAL4 lines from colleagues in the field. We now show images that indicate that its expression pattern is quite specific in Supplemental Figure 3.

3.      Young pdf5304 flies sleep more and have less success at mating. If sleep and mating success are related, would other types of wake or sleep promotion (pharmacological, thermogenetic) in young wild type flies also affect mating chances? Also, are pdf5304flies healthy, or can their reduced success at mating/increased sleep be explained as a sickness phenotype?

The literature increasingly suggests that sleep and waking impact mating. However, the relationship is complex. In our hands, the pdf5304 mutants are extremely healthy and have no difficulty mating. As seen in the manuscript, while they don’t mate much when sleep drive is high, they mate as well as control flies a day or two later indicating that the phenotype is not due to sickness.

Other specific comments:

1. Experimental details for assaying Day 0 behavior were included for Figure 2 but not Figure 1A-B. Please clarify method.

We thank Reviewer #3 for noticing this. The methods used in Figure 2 and Figure 1A-B, are in fact different. We have now added the following sentence to our description of Figure 1A-B in the main text: “As previously described, flies were collected and sexed using C02 anesthesia on the day they eclosed, placed into tubes and sleep was quantified during their first full day of adult life.”

2. For Figure 1G, it would be helpful to compare representative images between Day 0 and Day 5. Are there any other differences observed in PDFR expression between these ages? The findings in S1c-d suggest PDFR expression in the sLNv might decrease as well. If there are notable increases/decreases in PDFR levels in other groups, this should be quantified.

The data in Figure 1G is for Pdf-GAL4>UAS-GFP to simply show the circuit we are investigating. The image is thus not specific to PDFR levels. We haven’t found reliable differences in GFP between young and older animals. Reviewer #3 is correct, in noting that PDFR expression seems to decrease with age in the sLNvs as well. We have highlighted this observation regarding the sLNvs in the text. With respect to the Pdfr-myc data presented in Figure 1H, we focused exclusively on the lLNvs as these pertain directly to hour hypothesis. Studies are underway to determine other, novel neuronal groups that change their expression of PDFR following sleep disruption.

3. Statistical significance indicated in some graphs/panels but not others (eg. Figure 3A-C). Please also clarify statistical method used for Figure 3A-C.

We apologize for the confusion. The data in Figure 3A-C was evaluated using Kaplan-Meier analysis. We now report this explicitly in the Figure legend.

4. Previous reports have demonstrated a role for PDF/PDFR in adult sleep regulation (Parisky 2008, Chung 2009), including increased sleep in light phase in PDF/PDFR mutants. The authors should relate their current findings to these published data. A comparison in phenotypes between Day 0 and Day 5 for your backcrossed strains would be informative.

The loss of function experiments are problematic since adult flies do not typically express the PDFR. Thus, under baseline conditions, there is no sleep phenotype between experimental and control lines. Indeed, in figure 4B, we show that knocking down the Pdfr in adult flies does not alter sleep. Thus, we do not believe that evaluating sleep in adult pdfr5304 mutants will provide useful information relevant to the role of PDFR in the lLNvs.

5.  The methods relating to starvation related assays (Fig 4C,D; Fig 5A; Fig S3A) were not clearly described.

We have modified our Method section to better describe the starvation assays.

For starvation experiments, adult flies loaded into Trikinetics tubes containing 1% agar which provides water but not nutrients. Flies behavior was monitored until being evaluated for imaging or for survival experiments. The duration of starvation is noted in the text.

6. The format of Figure S3a is confusing given that some of the timepoints have an n of 0, thus PDF amplitude was not determined. Additional timepoints should be added and/or data should be binned differently.

We agree that the figure was confusing and have modified it and included it in the main Figures as requested by other Reviewers.

7. The methods should more clearly describe the differences between the 'sleep deprivation' and 'sleep restriction' protocols (Fig 5C).

The difference is how frequently the stimulus is applied to the fly. For sleep restriction, we activate the SNAP for 1 minute every 15 minutes. For sleep deprivation, the SNAP is activated continuously for the duration of the experiment. We have modified our Method section to state this explicitly.

Flies were placed into individual 65 mm tubes and a sleep-nullifying apparatus (SNAP) which mechanically disrupted sleep for one minute every fifteen minutes for 24-48 hours, which both reduced and fragmented sleep. For sleep deprivation, the SNAP was activated once every 8-seconds for the duration of the experiment.

8. Figure 5D, it is difficult to identify PDF+ MYC- large LNv in the baseline images shown. Higher resolution images should be provided with labels to indicate large LNv, ideally comparing a full cluster of large LNv for both baseline and sleep-restricted conditions.

The data shown for this construct is similar to that shown in other papers. We believe that this data is convincing but are willing to remove this at the editors request. Given our experience, it is unlikely that the images will look better with additional experiments.

9. nejire has previously been implicated in regulating CLK-CYC transcription activity- does nej RNAi affect pdfGAL4/ epac expression?

All of our imaging experiments include brains from an experimental group in the same dish as brains from a control group. There are no differences in the baseline levels of Epac-camps.

10. It is not clear which UAS-pdfr RNAi strain(s) were used.

We apologize. The line we used was the 42508 line; this has been added to the Method section.

11. "Receptor respecification is a form of plasticity that, like Hebbian and homeostatic plasticity, may be employed to alter circuit function in response to changing environmental demands [16]" Can the authors elaborate more on receptor respecification? I find the term a bit confusing - it suggests that one receptor is changed, respecified, to another receptor. But here it seems that a receptor appears in a circuit where it hasn't been previously observed. Also, the appearance of a new receptor should coincide with a change in presynaptic neurotransmitters. Can the authors speculate on which presynaptic inputs have changed?

As we mention in response to Reviewer #2, the term receptor respecification is used in a variety of contexts. Colloquially, the term is accurate with respect to our observations: “To specify again; to alter an earlier specification”. In our discussion, we note that the type of respecification we observe is different than that described in some of the more famous examples of receptor switching that occur during development.

We feel that our discussion provides the answers requested:

“The most common forms of respecification alter the polarity of the synapse to alter the function of the circuit [16]. Our data suggest an additional type of respecification in which an input pathway into a circuit can be turned on and off, without changing the sign of the synapse (excitatory/inhibitory). Presumably, turning on an input pathway may be a rapid first step to balance the impact of sustained activity in opposing circuits (e.g. sleep vs. wake). However, enhancing the activity of a circuit may create a positive-feedback loop which can destabilize the system and lead to adverse consequences.”

12. After the sleep restriction protocol that restricts fly sleep to bouts of 15 minutes, flies do not show increased rebound sleep. Is their sleep architecture (bout lengths, bout numbers) also unchanged?

Also, during the one minute sleep deprivation, how many times did the SNAP device move?

The SNAP moves every 10-s during the 1 minute of activation. We do not observe changes in average bout length of sleep bout number following restriction.

13. Fig 4C,D - survival curves are usually represented as Kaplan Meier plots and tested for significance using a logrank test. Can the authors reanalyze their data this way?

Thank you for the suggestion. The plots and statistics have now been updated.

Reviewer #4: Comments on "Sleep drive reconfigures clock circuitry…"

This is a promising paper with a set of interesting ideas woven together. It piggy backs onto the outstanding work of Kayser et al. (Sehgal lag) and more recently Matt Kayser from his own lab. They showed that very young flies, days 1-3 approximately after eclosion, sleep considerably more than more mature adults - like human babies. And the more recent work explores the molecular underpinnings of the initial observations. This paper takes off from these observations and finds that the expression of the famous PDF receptor (PDFR) in a well-studied group of circadian neurons is regulated in a similar fashion, namely, PDFR is expressed in the large ventral lateral neurons (l-LNvs) in very young adult flies but then disappears from these cells by day 5. This receptor is known to be expressed in many places in the fly brain including in the l-LNv's neighboring cells, the small ventral lateral neurons (s-LNvs), where it is present all the time in adult flies. The paper then goes on the explore the role of this receptor and how its expression is regulated. They argue that it is expressed in response to a number of stresses including (a particular version of) sleep deprivation, and they identify a transcription factor likely responsible for its synthesis as well as some receptors expressed in the l-LNvs that may be upstream of transcription factor activation.

Comments:

1) From the outset the paper is difficult to follow. The framing and beginning of the paper leads the reader to believe that PDFR expression in l-LNvs must contribute to sleep (since these very young flies sleep more), and yet it does the opposite. Indeed, these neurons have been shown to contribute to daytime activity and therefore would be expected to inhibit sleep. The presence of the receptor causes them to do this only more so it seems to me. A clear definitive sentence at the end of the first paragraph of the Results saying something like this and leading into paragraph 2 would be helpful.

We thank the Reviewer for this suggestion. We have now modified the last sentence to read: Together these data indicate that the PDFR is transiently expressed in wake-promoting lLNvs in young flies to support waking when sleep-drive is highest.

2) The paper relies heavily on the han (PDFR) mutant strain and I think a single RNAi. Also, the key figure 2A should be done in parallel with the pdf01 mutant strain. The results should be very similar, no?

With respect to PDFR functioning, a growing literature indicates that the pdf01 mutant frequently does not fully recapitulate what is seen with the pdfr-mutants. One reason for the lack of overlap in phenotypes is that the PDFR is promiscuous. In any event, when viewed together, pdfr-WT, pdf-RNAi, pdfr5403 all present a coherent picture.

And Figure 2A is missing the data from ZT0-ZT4. Nonetheless, it looks like the WT has morning anticipation here but not in Fig. 1. How come?

We thank reviewer #4 for catching an error in nomenclature. The flies in Figure 1 were collected at various times during their day of eclosion while being exposed to C02. They were then placed into tubes and their sleep was quantified beginning at lights on the following day. Since the flies eclosed at different point during their day of eclosion, we refer to their first full day after eclosion as Day 0. However, in Figure 2A, the flies were pre-loaded as larvae so that we could capture the entirety of their behavior without having to expose them to C02. Thus the legend on Figure 2A should read Day of Eclosion not Day 0. We have corrected the figure legend and added a description of how we quantified sleep for Figure 1A,B. We believe that this also explains the discrepancy in anticipation….however, one must be cautious using sleep plots to define morning or evening anticipation. The sleep plots shown display the data in1 h long bins and are not designed to calculate anticipation.

And I'd like to see the % sleep change at each ZT value as well as activity plots and their % change at each ZT.

The salient features of behavior are accurately expressed in Figure 2A. Since the flies have just eclosed, there is no %change to calculate. In any event, this data is as close to raw data as one can get and is thus the most informative way of presenting the data. For Figure 1A, these data replicate previous experiments and is the standard way of presenting the data. Note the data in figure 1A is a within subject design such that the reader can quickly see how sleep changes over days.

Since the l-LNvs have been shown to contribute to daytime activity in LD, a contribution to activity and therefore some inhibition of sleep is not too surprising - as mentioned above. So is this really specific for some "wake-maintenance zone?".

The wake-maintenance zone has been defined in previous studies based upon the attenuated sleep rebound observed when flies are released into recovery shortly before lights out. The methodology and interpretation used in flies is similar to that used to define the WMZ in mammals. The data presented in Figure 2B, indicates that the l-LNvs are not able to promote waking at other times of day in very young flies.

And to what extent is this attributable specifically to this PDFR expression; two RNAis here are essential. And this is an issue throughout the paper, where single RNAs appear to be frequently sufficient to draw a conclusion.  (Have at least two RNAis been used to validate the transcription factors identified near the end of the paper?)

As mentioned above, pdfr-RNAi is now a well characterized tool that has been validated many times throughout the literature. When viewed together, the pdfr-RNAi, pdfr-WT and han5304 data present a coherent picture.

As is common for most mini-screens, only a single RNAi line is used to initially look for phenotypes. Additional validation only occurs after a phenotype is overserved. As seen in Figure 6, we validated the nejire results using two RNAi lines, and completed a gain-of-function experiment that exhibited complementary behavior with RNAi. Although we had a phenotype with SUUR, we only had one RNAi line at the time and so focused on nejire instead.

3) tPDF and uas-PDFR could also be expressed in l-LNvs (specific gal4 is much better than c929) and in a pdf01 background. This would make the expression data more convincing. This would be much easier for the authors than a direct PDFR RNA assay from purified l-LNvs, which is another alternative.

There are certainly additional tools that can be used. However, we expressed UAS-Pdfr-wt using a split-GAL4 line and found that it increased waking during the wake-maintenance zone. We have replicated these data using *Pdfr^5304^;PDF-GAL4* (Figure S2B) and a ‘wild-type’ *Pdf-GAL4* driver (data not shown). Together with the physiology showing the PDFR is expressed in the lLNvs following sleep deprivation, sleep restriction and starvation, these data present a very coherent picture.

4) The numbers for live imaging are extremely low (5 neurons? That's maybe 2 brains). How many neurons react (1-4) and how reproducibly?

We apologize for our error in nomenclature. The values presented are for Regions of Interest (ROI) in each hemi-segment of the fly brain. An RO1 consists of 2-4 l-LNv neurons. We evaluated a minimum of 4 separate brains. The text has been corrected accordingly.

5) In Fig. 3B, why are the parental controls so low compared to CantonS in Fig. 3A? If statistics are done on all of the data in Fig. 3A and 3B together, would the PDFR mutant statistically differ from the parental controls of the RNAi experiment?

The data shown in Fig. 3A and Fig. 3B are independent experiments conducted at separate times on separate genotypes. These experiments were replicated 2-3 times over several months. In any event, the experiment displayed in Fig. 3B is self-contained and the comparison is made for the experimental flies with their precise parental controls reared on the same food etc.; this is the only appropriate comparison. Similarly, the data shown in Figure 3A compares mutant flies to their genetic controls. Any additional comparison with any other dataset is misleading and prone to error.

6) How often were the mating success experiments done? No error bars indicate n=1 (M&M also not clear). Also, the data are not very convincing, the WT controls in Fig. 3B are probably not significantly different from the han mutant in Fig 3A. How do the authors explain the index of 0? This suggests too many constructs rather than an effect of PDFR knockdown (pdf01 and han mutants are not sterile). The PDFR rescue should be done with lLNv-GAL4.

All of the mating experiments were done in triplicate. Since the outcome is binary (offspring or no offspring), we conducted a chi-square and the results are highly significant. The data in Figure 3B were conducted using the yeast UAS/GAL4 system where experimental animals are compared to their parental controls. The data in Figure 3A evaluates wild-type flies compared to the outcrossed mutant. We are not aware of any rationale that would support comparing data from such dramatically different experiments to each other (e.g. 3A vs 3B).

7) Labeling of images in Fig 5D is inverted.

We thank the Reviewer for catching this. The labels are now corrected.

8) How can a knockdown of dopamine receptors have no effect on the reactivity of the cell to dopamine? Once again a second RNAi is necessary to make this surprising claim.

Given that all 4 DA receptors are expressed in the l-LNvs this observation is not surprising at all. Indeed, a recent publication has also found that the lLNvs respond to DA after individual DA receptors are knocked down (DOI: 10.1523/JNEUROSCI.1488-20.2020). As with pdfr-RNAi, all of the RNAi lines for the DA receptors are well validated. More importantly, we have validated all these lines further, (i.e. using 2 RNAi lines) for our learning experiments demonstrating that these lines continue to be as effective as the literature suggests.

9) I don't think the lifetime experiments (Fig. 4) are very compelling. I think of this more as manipulating activity than sleep. It makes sense that starving a more hyperactive fly is worse than a less active fly. Can this be done in parallel with another hyperactive strain?

We have previously shown that similar amounts of waking activity induced by either sleep deprivation or starvation result in dramatically different outcomes as measured by survival, sleep homeostasis and learning. That is, the relationship between sleep and starvation is not as straightforward as it might seem at first glance. Regardless, the main question isn’t about activity or sleep; it is about how the expression of the pdfr impacts adaptive behavior. When flies express the pdfr-WT in the l-LNvs, they die sooner during starvation. In contrast, when flies are blocked from expressing the pdfr in the l-LNvs, they live longer during starvation.

10) The expression by different modalities is for sure interesting. The reader should learn if only this very special way to sleep deprive upregulates receptor expression or if a more traditional deprivation strategy can do the same thing.

We agree with Reviewer #4 that the different ways of disrupting sleep will provide additional tools for studying sleep regulation and function. With that in mind, both sleep restriction and sleep deprivation result in the pdfr being added to the l-LNvs in adults (Figure 5C). However, our data indicate that sleep restriction is a much milder intervention and may turn out to be more effective in teasing out specific and non-specific effects of sleep disruption.

11) Can we see the time of day data that underlies the decreased sleep (and presumably increased activity) in Fig. 5F, the sleep and activity profiles?  Are the flies just generally more active, or is there some interesting time of day information? Perhaps the upregulation is just a stress-induced flight or fight response? A bit like enhancing foraging in response to starvation.

The data in Figure 5F shows the %sleep recovered following sleep loss. All flies showed an increase in sleep during recovery; the dcr2;C929>UAS-Pdfr-RNAi flies showed a larger increase in sleep during recovery.

12) What's the sleep phenotype in the manipulations of Fig 6? Do nej knockdown flies phenocopy the absence of PDFR?

Please see response to Reviewer #2.
