# Supplementary material for: Sleep drive reconfigures wake-promoting clock circuitry to regulate adaptive behavior
Source: PLoS Biol. 2021 Jun 30;19(6):e3001324. doi: 10.1371/journal.pbio.3001324 (PMC8277072; doi:10.1371/journal.pbio.3001324)
Supplement: Attachment — Submitted filename: Response to Reviewers.docx [file pbio.3001324.s018.docx]

REVIEWS:

Reviewer #1: The authors have responded directly to the majority of the (all minor) concerns I expressed in my first review. I have no further concerns. It's an interesting, clear, and significant study.

We thank Reviewer #1 for their help in improving the manuscript.

Reviewer #2: Most of my concerns have been addressed. There is one lingering issue. I had realized that figure 2E showed sleep during WMZ. The value for controls in this panel are around 25-40, but on panel 2C CS shows a value of about 85. Those are rather big differences between different control flies. Where is this variability coming from? Is this a genetic background issue? Was pdfr-5304 backcrossed to CS? Controls for genetic backgrounds are good within figures, and a genetic rescue for the pdfr mutant phenotype is presented, so this does not seem to be a major concern, but some explanations are needed.

The two panels show sleep in different genotypes from experiments conducted at different times and thus should not be compared directly. Since 1997, when we began evaluating sleep in flies, we have observed that independent cohorts for any given genotype can, at times, display a fair amount of variability. We have very strict protocols to ensure that we set up crosses using flies of the same age and that we use the same number of flies for each and every cross (8 females, 4 males). All flies are maintained on a 12:12 LD schedule at 50% humidity during rearing and testing. The flies being tested are evaluated on the same diet as they are reared. We have shared these observations with other *Drosophila* sleep neurobiologists at meetings but no consensus about what type of environmental factors might contribute to this variability has reached consensus. To control for these unknown environmental variables, we always include contemporaneous controls for each experiment.

Pdfr-5304 flies were backcrossed to Cs flies.

Minor: The last paragraph of the results on p.9 contains a fragment of sentence that needs to be removed.

We thank Reviewer #2 for catching this mistake. The fragment has been removed.

I was also asked to look closely at the responses to the comments made by reviewer#4. Again, I think the authors did a good job responding to these comments.

1)	One issue that was brought up not just by reviewer #4 but also reviewer #1 is whether pdf0 phenocopies pdfr. This is a fair comment, and I agree with the authors' reply that pdf and pdfr mutants do not necessarily phenocopy each other, due to promiscuity of the pdfr receptor. However, this will be a question in the mind of many readers, and I would suggest that authors show pdf0 mutant data if they have it.

We now include data on the *pdf^01^* mutant in Supplemental Figure S4. We obtained the *pdf^01^* mutants and their genetic controls directly from Dr. Paul Taghert. We conducted two replicates and show a representative result in Fig. S4. In contrast to pdfr*^5304^* mutants that maintain sleep during starvation, *pdf^01^* mutants lose as much sleep as background controls. However, starvation does not increase waking activity in *pdf^01^* mutants and they survive longer during starvation Together these data indicate that the ability of *pdf* to influence adaptive behavior during starvation extends beyond modifying sleep time. These results are now included in the main text.

2)	I would like to comment on the issue of the need for two RNAis, which was brought up by reviewer #4 and #1. For pdfr phenotypes, I feel that a single RNAi phenocopying the pdfr mutant is sufficient, particularly since the authors can rescue this mutant. For the screen, when only one RNAi line is used, some words of caution would be worth adding.

Reviewer #3: The authors have addressed my major comments--I have one remaining issue that I think would be beneficial for them to address. The authors have nicely clarified in their comments to the reviewers the definition of respecification. I suggest that they provide this level of clarity when they first introduce the concept in the paper and make clear the distinction with other examples in the literature.

We thank Reviewer #3 for this suggestion and have included our definition of respecification in the introduction so that the distinction between our findings and other examples in the literature are clearly stated from the beginning.

With regards to reviewer 4's comment on pdf01, the authors make note of differences between pdf01 and pdfr mutants and their frustration with pdf01. I do think it would be valuable to include pdf01 data even if negative as it would highlight those differences and provide some insight into the relevant ligand for pdfr. Nonetheless the authors should make reference to the differences between pdf01 and pdfr mutants when discussing potential mechanisms by which pdfr might be functioning, i.e., pdfr may not be necessarily be working via PDF activation.

We have now included data on the *pdf^01^* in response to Reviewer #2, see above.
