# Supplementary material for: Sleep drive reconfigures wake-promoting clock circuitry to regulate adaptive behavior
Source: PLoS Biol. 2021 Jun 30;19(6):e3001324. doi: 10.1371/journal.pbio.3001324 (PMC8277072; doi:10.1371/journal.pbio.3001324)
Supplement: Attachment — Submitted filename: Response to Reviewers.docx [file pbio.3001324.s019.docx]

**Reviewers: We think that the revised manuscript has, for the most part, addressed the concerns of the reviewers in the last round of review. However, we agree with the reviewers that it would be important to tone down statements regarding the RNAi screen on cell surface receptors, given that these are based on experiments with one RNAi line for each gene. Without using more than one RNAi line or transgene rescue, we think that you should not conclude positive effects nor negative effects from this screen, as there is the possibility that these effects were caused by off-target interactions or due to ineffective knockdown. The last paragraph of the results section should therefore be substantially revised, or even removed.**

We have substantially modified the last paragraph of the results section. We now simply describe the experiments underlying the data shown in Figure 6 E,F. We also specifically note that the data only represent 1 RNAi line for each receptor and thus should be viewed cautiously. We hope that these modifications satisfy the reviewer as we believe that data from screens such as this are extremely valuable to the reader. From a personal perspective, I have successfully followed up on similar types of data that I found in the literature. It should also be noted that some of the RNAi lines shown in Figure 6 have been also validated in the literature. My lab has extensive experience using these RNAi lines for Dop1R1, Dop1R2 D2R, Dopecr, and Npfr but unfortunately only for unrelated phenotypes. Nonetheless, we only used one RNAi line and we agree with the reviewers that it is best if this fact is highlighted specifically.

The last paragraph now reads:

Finally, we asked whether similar mechanisms were used by young and mature adults for the activation of *nejire*. To identify cell surface receptors that might interact with *nejire*, we once again consulted a database of genes known to be enriched in the LNvs [50]. We then conducted a targeted RNAi screen to evaluate PDF sensitivity in young flies and mature-adults following sleep restriction (Fig. 6e,f and Fig. S6C, D). Given that we only evaluated 1 RNAi line to evaluate each receptor, these results should be viewed cautiously. Nonetheless, these data suggest the possibility that *DopEcR*, *Dop1R2*, and *InR* may play a role in the respecification of the PDFR in both young flies and mature adults during sleep restriction.
